# Phylogenetic relations among Mexican phlebotomine sand flies (Diptera: Psychodidae) and their divergence time estimation

**DOI:** 10.1371/journal.pone.0287853

**Published:** 2023-06-29

**Authors:** Yokomi N. Lozano-Sardaneta, Jesús A. Díaz-Cruz, Vicente Viveros-Santos, Sergio Ibáñez-Bernal, Herón Huerta, Carlos F. Marina, Pedro C. Mis-Ávila, Maribel Martínez-Burgos, Jorge A. Torres-Monzón, Víctor Sánchez-Cordero, Ingeborg Becker

**Affiliations:** 1 Centro de Medicina Tropical, Unidad de Medicina Experimental, Facultad de Medicina, Universidad Nacional Autónoma de México, Ciudad de México, México; 2 Facultad de Ciencias, Universidad Nacional Autónoma de México, Ciudad de México, México; 3 Colección Nacional de Peces, Departamento de Zoología, Instituto de Biología, Universidad Nacional Autónoma de México, Ciudad de México, México; 4 Centro Regional de Investigación en Salud Pública, Instituto Nacional de Salud Pública (CRISP-INSP), Tapachula, Chiapas, México; 5 Instituto de Ecología, A. C. (INECOL), Red Ambiente y Sustentabilidad, Xalapa, Veracruz, México; 6 Laboratorio de Entomología, Instituto de Diagnóstico y Referencia Epidemiológicos ‘Dr, Manuel Martínez Báez’, Ciudad de México, México; 7 Servicios Estatales de Salud de Quintana Roo, Departamento de Enfermedades Transmitidas por Vector y Zoonosis, Chetumal, Quintana Roo, México; 8 Departamento de Zoología, Instituto de Biología, Universidad Nacional Autónoma de México, Ciudad de México, México; Federal University of Mato Grosso do Sul, BRAZIL

## Abstract

Phlebotomine sand flies (Diptera: Psychodidae: Phlebotominae) have biological relevance as vectors of several pathogens. To ensure periodic entomological monitoring it is necessary to have efficient and accurate tools for an adequate taxonomic identification. There are only few studies on phylogenetic analyses of phlebotomine sand flies from Neotropics, based mostly on morphological and/or molecular data, which makes the delimitation of intra- and interspecific variability of species challenging. Here we generated new molecular information on sand fly species distributed in endemic areas of leishmaniasis in Mexico, using mitochondrial and ribosomal genes, and incorporating morphological information available. Specifically, we established their phylogenetic relationships, and estimated their divergence time. Our study provides molecular information for 15 phlebotomine sand fly species from different areas of Mexico, contributing to the genetic inventory and phylogenetic relations among Neotropical species of the subfamily Phlebotominae. Mitochondrial genes proved to be suitable markers for the molecular identification of phlebotomine sand flies. However, the incorporation of additional nuclear gene information could increase the significance of phylogenetic inferences. We also provided evidence about a possible divergence time of phlebotomine sand fly species, supporting their presumable origin in the Cretaceous period.

## 1. Introduction

Phlebotomine sand flies (Diptera: Psychodidae: Phlebotominae) are of medical-veterinary relevance due to their role as vectors of *Leishmania*, *Bartonella* and some arboviruses [1]. To ensure entomological monitoring and provide basic information promoting surveillance programs for vector-borne diseases, it is necessary to have efficient and adequate tools for a reliable taxonomic identification.

The taxonomic identification of phlebotomine sand flies is mostly based on morphological and morphometric characters, with the use of specialized taxonomic keys. A total of 1,060 phlebotomine sand fly species have been described worldwide, of which 556 species are present in the New World [2]. Previously, the phlebotomine sand flies distributed in the Americas were classified into three genera, being the genus *Lutzomyia* França and *Brumptomyia* França & Parrot, the most widely distributed [3]. Recent advances in the taxonomic classification based on phylogenetic analyses of morphological characteristics, allowed a more detailed classification for phlebotomine sand flies, recognizing at least 23 genera that have now been widely adopted by researchers of the Phlebotominae subfamily [4]. However, the morphological identification remains limited for some species, since it requires skills and expertise to manipulate these small specimens, which increases the risk of damaging key morphological structures. These can be easily damaged during the mounting process on a coverslip, which makes the morphological identification even harder [4–6]. Furthermore, some species are recognized as cryptic, showing high phenotypic plasticity, intraspecific polymorphism, and even morphological similarity between females in various genera or subgenera, lacking distinctive morphological characters to enable their conventional identification [7].

Despite the clinical importance of phlebotomine sand flies, few studies have been conducted to assess an integrative taxonomy with the use of morphological and/or molecular data [7–11]. Taxonomic proposals based on morphological data have been used as a framework for most inclusive phylogenies [3, 11]. Yet, molecular data are still lacking for most phlebotomine sand flies (adult and immature stages), since only 37% of them have molecular information, and not all of the studies have been performed using the same genes, nor has the same specimen been sequenced for multiple markers [2, 8, 12]. The only genera with abundant molecular records include *Phlebotomus*, *Nyssomyia*, *Psathyromyia* and *Psychodopygus* [12].

Molecular methodologies use different kinds of markers to explore different taxonomic questions at different levels such as: (1) ribosomal gene sequences for the relations within the family or some genera (18S rDNA) [13], (2) mitochondrial genes for their faster rate of evolution and low recombination, which allows to discriminate between species, even if they are cryptic or closely related species [cytochrome b (*cytb*), NADH Dehydrogenase 4 (ND4), and cytochrome oxidase subunit 1 (*COI*)] [14–16], and (3) nuclear genes to resolve intraspecific and subgeneric relationships [the Internal Transcribed Spacer (ITS1) and nuclear Elongation Factor 1-alpha genes (EF-1a)] [5, 7]. Therefore, interspecific relations of sand flies remain unknown in many cases, and complementary studies are still lacking in several countries. Solving the phylogenetic relations among Neotropical phlebotomine sand flies is relevant for: (1) testing the current taxonomy based on morphological characters; (2) reconstructing the geographic origin of each lineage; (3) identifying cases of speciation, cryptic diversity and new species; and (4) estimating the timing of historical events [8]. For that reason, a comprehensive Phlebotominae phylogeny will shed light on phlebotomine sand fly biology and systematics, allowing to validate morphological differences between species, as well as to explore interspecific and intraspecific relations [8].

In Mexico, 50 species of phlebotomine sand flies and two fossil species have been described in at least 24 states, of which Chiapas (36 spp.), Quintana Roo (24 spp.) and Veracruz (23 spp.) hold the highest species richness of phlebotomine sand flies, along with a high number of human cases of leishmaniasis [17]. However, only around 20% (11 species) of the known Mexican sand flies possess DNA barcodes *COI* for molecular identification [14, 15], and only one study has analysed the genetic diversity of *Lutzomyia cruciata* using *cytb* [16]. Thus, the genetic information for the molecular identification of Mexican sand fly species remains limited, which difficult the delimitation of intra- and interspecific variability of species. This study now provides new molecular information on phlebotomine sand flies distributed in endemic areas of leishmaniasis in Mexico, using mitochondrial and ribosomal genes. Molecular information was combined with the available morphological information on these species, to establish their phylogenetic relations with regard to other specimens of the same species and genera, regardless of their geographic distribution. Furthermore, their divergence time was estimated, including morphological and temporal information of fossil species. We also tested whether the use of *COI*, *cytb* and 18S rDNA genes are useful as complementary tools to aid traditional taxonomy for identification at a specific level.

## 2. Material and methods

### 2.1 Specimen samples

**Fig 1:**
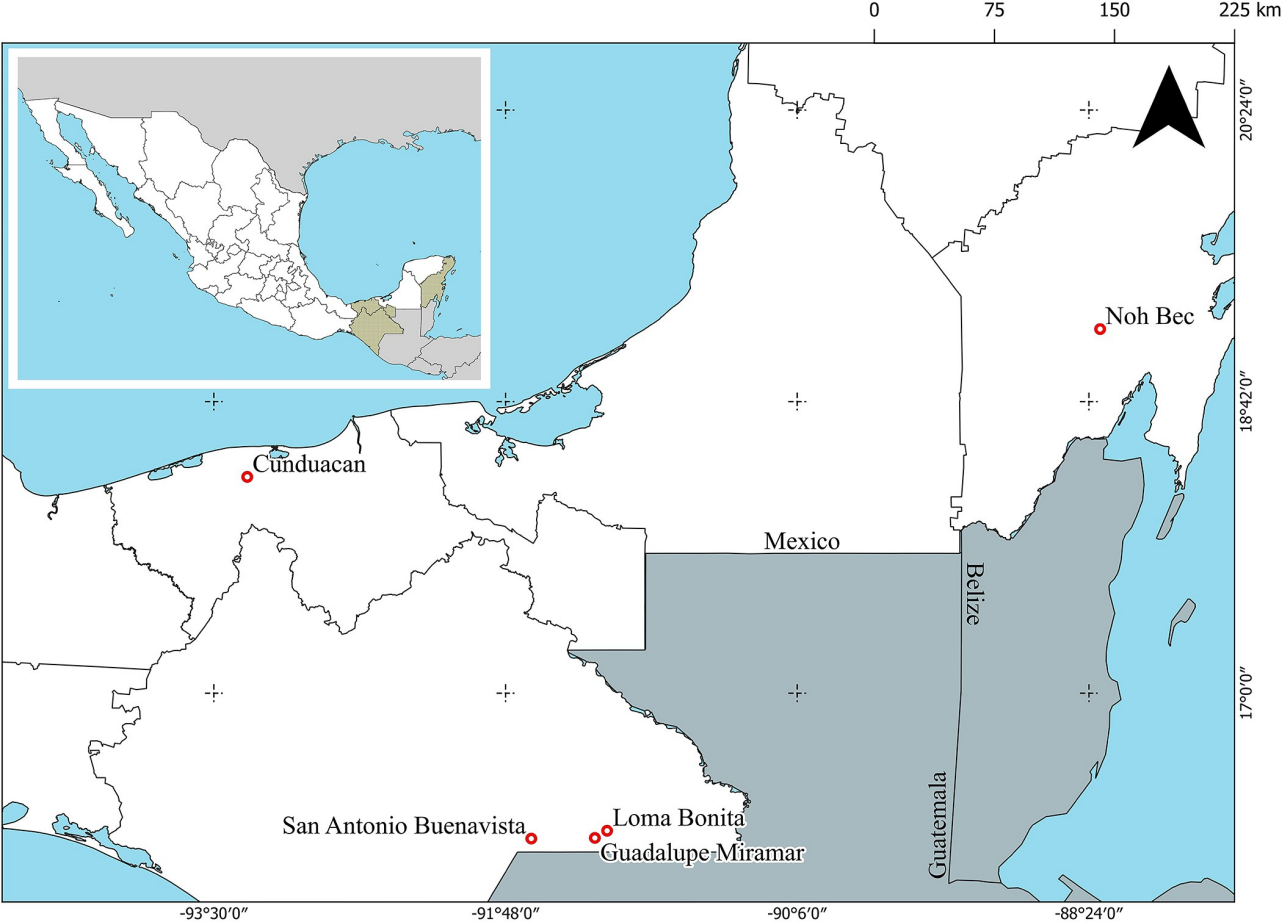
Map depicts the geographical locations (red dots) of the samples collected in the states of Chiapas, Quintana Roo, and Tabasco, Mexico. Green coloured states correspond to those where samples were collected.

We analysed DNA of phlebotomine sand fly species previously collected in endemic areas of cutaneous leishmaniasis in the states of Chiapas, Quintana Roo and Tabasco, Mexico. The sampling localities were: 1) San Antonio Buenavista, Chiapas (16° 09’ 08” N; 91° 38’ 58.9” W; 1,380 m.a.s.l.); 2) Guadalupe Miramar, Chiapas (16° 09’ 22.6” N; 91° 16’ 45.2” W; 432 m.a.s.l.); 3) Loma Bonita, Chiapas (16° 11’ 53” N; 91° 11’ 88.4” W; 210 m.a.s.l.), during the period April 2009 to March 2011 [18]; 4) Noh Bec, Quintana Roo (19°02’ 30” N; 88°13’ 33” W, 30 m.a.s.l.), during the period November 2021 to May 2022; and 5) Huimango, Cunduacan, Tabasco (18° 08′ 46ʺ N, − 93 10′ 50ʺ W; 6 m.a.s.l.), during October 2019 [19] (Fig 1). The specimens had been collected with CDC light traps (Mod. 512).

### 2.2 Morphological identification

The taxonomic identification of phlebotomine sand fly species was done according to the dichotomous key proposed by Ibáñez-Bernal [20, 21], and the phylogenetic classification proposal of Galati [11], examining the head, the last segment of the abdomen (male and female terminalia) and the wings. The remaining parts of the body (thorax, legs and the first segment of the abdomen) were used for the molecular analysis. These structures were preserved in alcohol at 70% and refrigerated at -20°C for DNA extraction. We used the abbreviation system proposed by Marcondes [22].

We optimized a semi-permanent mounting on a glass slide, using a modified Hoyer´s medium [23]. The medium was prepared using pure glycerine, distilled water and Arabic gum in a proportion 1:2:2. During the mounting process, we put a drop of the medium on the slide and added a drop of sodium hydroxide (NaOH) at 8% and a drop of the pigment benzalkonium chloride at 0.13% (for a better and easier visualization of important structures, such as ascoids). The structures were placed in the medium, and dried at room temperature for 15–20 min. The specimens were visualized with a compound microscope Carl Zeiss model Primostar 3 with objectives 10X and 40X and the structures were photographed with a camera Axiocam 208 color (Software Zen Lite). The pictures obtained were visualized and edited in Adobe Photoshop CS5 software (S1 Fig). The slides are deposited in the Centro de Medicina Tropical, UNAM, and Instituto Nacional de Salud Publica (CRISP-INSP).

### 2.3 DNA extraction, amplification, and sequencing

DNA was extracted from phlebotomine sand fly abdomen, thorax and legs, using published protocols [16, 24]. We amplified a fragment of ~ 600 bp for the mitochondrial gene cytochrome oxidase subunit 1 (*COI*) using the primers LCO1490 (5’-GGT CAA CAA ATC ATA AAG ATA TTG G-3’) and HCO2198 (5’-TAA ACT TCA GGG TGA CCA AAA AAT CA-3’) [25]. The other mitochondrial gene used was cytochrome b (*cytb*). We amplified a fragment of ~ 365 bp using the primers 11226 (5’-GAA TGA TAT TTT TTA TTT GC-3’) and 11587 (5’-CTT ATG TTT TCA AGA CAT ATG C-3’). Finally, we amplified a fragment of ~ 450 bp for a conserved region of the 18S rDNA gene, using the primers Lu.18S rRNA-1S (5’-TGC CAG TAG TTA TAT GCT TG-3’) and Lu.18S rRNA-1R (5’-TTA CGC GCC TGC TGC CTT CC-3’) [13]. The PCR conditions for the *COI* gene were performed with an initial denaturation at 94°C for 10 min, followed by 35 cycles of 94°C for 30 sec, 50°C for 30 sec, 72°C for 45 sec, and a final extension at 72°C for 5 min. For the *cytb* gene, the PCR conditions were performed with an initial denaturation at 95°C for 5 min, followed by 35 cycles of 95°C for 60 sec, 40°C for 60 sec and 72°C for 90 sec and a final extension at 72°C for 10 min. For the 18S rDNA gene we used an initial denaturation at 95°C for 2 min, followed by 30 cycles of 95°C for 60 sec, 55°C for 60 sec and 72°C for 60 sec and a final extension at 72°C for 10 min. The reaction mixture was prepared in a final volume of 25 μl containing 12.5 μl GoTaq® Green Master Mix 2X Promega Corporation (Madison, WI, USA), 1 μl of each primer (100 ng each), 5 μl DNA template (~50 ng/μl), and 5.5 μl nuclease-free water. The PCR reactions were performed in a Veriti 96 Well Thermal Cycler (Applied Biosystems^TM^, Termo Fisher Scientific, USA). The amplified products were analysed by electrophoresis in 2% agarose gels stained with 0.4 μL of Midori Green Advance (Nippon genetics). PCR products were purified and sequenced at the Laboratorio de Secuenciación Genómica de la Biodiversidad y de la Salud, Instituto de Biología, UNAM.

### 2.4 Phylogenetic analysis

The electropherograms were visualized and edited in the software Chromas version 2. 6. 6 (http://technelysium.com.au/). Each sequence was compared with all the sequences available at NCBI database, using BLASTn (https://blast.ncbi.nlm.nih.gov/Blast.cgi) as a preliminary confirmation. The obtained sequences were deposited in the GenBank database; the accession numbers are available in Table 1. The retrieved sequences were aligned with other sequences of the *COI*, *cytb* and 18S rDNA genes available in GenBank, using MEGA X [26]. First, we analysed the sequences obtained for each gene individually using a Maximum Likelihood (ML) reconstruction performed in MEGA X, with 1, 000 bootstraps. The sequences of the *cytb* gene were analysed with the General Time Reversible+ Gamma distribution substitution model (GTR+G), showing a Bayesian information criterion (BIC) score of 5767.106. The 18S rDNA sequences were analysed with Tamura 3 parameters + Gamma distribution + Invariant sites substitution model (T92+G+I), showing a BIC score of 2326.915. For the *COI* gene, we used the General Time Reversible+ Gamma distribution+ Invariant sites substitution model (GTR+G+I), showing a BIC score of 6218.578. Since the *COI* gene is useful for genetic barcodes, the aligned sequences were collapsed into unique haplotypes using the FaBox online toolbox for FASTA sequences [https://users-birc.au.dk/palle/php/fabox/] [27]. The number of haplotypes (H), polymorphic sites (s), nucleotide diversity per species (π), and haplotype diversity (Hd) were calculated in DnaSP v5.10 [28]. Genetic pairwise distances were estimated using the Kimura-2-parameter substitution model (K2P) in MEGA X. The barcode gap (gap between intra and interspecific genetic distances) graph was calculated using ggplot2 and the Easy Ggplot package in R environment [29].

**Table 1:** List of phlebotomine sand fly species collected from the states of Chiapas, Quintana Roo, Tabasco in Mexico and analysed using molecular and morphological data.

Species	N	Sex	H	Locality	State	Intraspecific distances‡	Accession numbers *COI*	Accession numbers *cytb*	Accession numbers 18S rDNA
*Bichromomyia olmeca* (Vargas & Díaz-Nájera)	3	♀	1 1	GM NB	Chiapas Q. Roo	0.19/0.3/0.0	OP784392 OP784393	OQ343430 OQ343447 OQ343449	OQ341288 OQ341314 OQ341316
*Brumptomyia mesai* Sherlock	4	♀(1) ♂(3)	1	NB	Q. Roo	0.0/-/0.0	OP784394 OP784395 OP784396	OQ343446	OQ341312 OQ341313
*Dampfomyia beltrani* (Vargas & Díaz-Nájera)	4	♀	1	LB NB	Chiapas Q. Roo	0.0/1.23/0.0	OP781331 OP781332	OQ343439 OQ343440	OQ341300 OQ341301 OQ341302
*Dampfomyia deleoni* (Fairchild & Hertig)	7	♀(5) ♂(2)	1	LB NB	Chiapas Q. Roo	0.0/0.93/0.0	OP784405 OP784406	OQ343438 OQ343444 OQ343445 OQ343448	OQ341295 OQ341308 OQ341309 OQ341310 OQ341315
*Dampfomyia delpozoi* (Vargas & Díaz-Nájera)	1	♀	-	GM	Chiapas	-/-/-	-	-	OQ341292
*Dampfomyia leohidalgoi* (Ibáñez-Bernal, Hernández-Xoliot & Mendoza)	1	♀	-	LB	Chiapas	-/-/-	-	-	OQ341297
*Lutzomyia cruciata* (Coquillett)	10	♀	2 1	SAB,GM NB	Chiapas Q. Roo	0.28/2.20/0.24	OP784399 OP784400 OP784401 OP784402 OQ325337	OQ343428 OQ343432 OQ343437 OQ343441 OQ343442 OQ343443	OQ341284 OQ341287 OQ341290 OQ341293 OQ341305 OQ341306 OQ341307
*Micropygomyia chiapanensis* (Dampf)	1	♀	-	Hu	Tabasco	-/-/-	-	OQ343452	OQ341319
*Nyssomyia ylephiletor* (Fairchild & Hertig)	5	♀	3	GM	Chiapas	0.26/0.93/-	OP784407 OP784408 OP784409 OP784410 OP784411	OQ343427 OQ343429 OQ343434	OQ341285
*Pintomyia ovallesi* (Ortiz)	3	♀	2	SAB,LB	Chiapas	0.0/0.30/0.0	OP784403 OP784404	OQ343426 OQ343431 OQ343433	OQ341286 OQ341289
*Psathyromyia carpenteri* (Fairchild & Hertig)	2	♀	1 1	LB NB	Chiapas Q. Roo	0.0/-/0.0	OP784390 OQ325338		OQ341296 OQ341303
*Psathyromyia maya* Ibáñez-Bernal, May-UC & Rebollar-Tellez	2	♀	1	NB	Q. Roo	0.0/-/-	OP784397 OP784398	-	OQ341311
*Psathyromyia shannoni* (Dyar)	3	♀(1) ♂(2)	1 1	SAB NB	Chiapas Q. Roo	1.26/0.61/0.0	OP784412 OP784413 OP784414	OQ343450 OQ343451	OQ341317 OQ341318
*Psathyromyia texana* (Dampf)	1	♀	1	NB	Q. Roo	-/-/-	OP784391	-	OQ341304
*Psychodopygus panamensis* (Shannon)	7	♀	2 1	GM NB	Chiapas Q. Roo	0.13/0.30/0.48	OP784415 OP784416 OP784417	OQ343435 OQ343436 OQ343453 OQ343454 OQ343455	OQ341291 OQ341294 OQ341298 OQ341320 OQ341321
**15 species**	**54**	**-**	**22**	**5** *	**3**	**-**	**32**	**30**	**37**

N = number of specimens analysed for obtaining sequences. H = haplotypes using only the *COI* gene.

* GM = Guadalupe Miramar, LB = Loma Bonita, SAB = San Antonio Buenavista, NB = Noh Bec, Hu = Huminago. Q. Roo = Quintana Roo.

‡ the intraspecific distances of *COI*/ *cytb*/ 18S rDNA genes, only using our sequences.

Furthermore, we downloaded GenBank sequences of *COI*, *cytb*, and 18S rDNA for the same species or genera obtained in this study. In the case where the sequences available in GenBank were from the same study, same location and the same species, we randomly selected some sequences. The included genera distributed in Mexico and in other Neotropical areas were: *Bichromomyia* (*Bi*.), *Brumptomyia* (*Br*.), *Dampfomyia* (*Da*.), *Evandromyia* (*Ev*.), *Lutzomyia* (*Lu*.), *Micropygomyia* (*Mi*.), *Nyssomyia* (*Ny*.), *Pintomyia* (*Pi*.), *Pressatia* (*Pr*.), *Psathyromyia* (*Pa*.), and *Psychodopygus* (*Ps*.), whereas species of the genus *Phlebotomus* of the Old World were selected as out-group, given its closeness to the in-group taxa. The GenBank repository contains numerous sequences of the *COI*, *cytb* and 18S rDNA genes for several phlebotomine sand fly species of South America. However, when we analysed the information, many of these sequences showed a mismatch with the amplified fragments and sequences of our study. Therefore, we excluded only the taxa sequences that showed extremely discordant relations to our sequences. This problem was observed mainly with 18S rDNA sequences, probably because this gene is represented in the genome by a large array of non-identical paralogues [8].

The final alignment files were concatenated in the program Mesquite v 3.7 [30]. Phylogenetic analyses were performed using two combinations: (i) using only genetic data (mitochondrial and ribosomal genes), and (ii) using molecular sequences and discrete morphological data. We used Maximum likelihood as optimality criterion for the phylogenetic reconstructions and the analyses were performed in IQtree v 2.2 [31], using ModelFinder [32] to determine the model that best fitted our data. We selected the model according to the BIC score, using a partition scheme for the coding sequences. The selected models for the concatenated genes included *COI*: SYM+I+G4, *cytb*: GTR+F+G4 and 18S rDNA: GTR+F+G4. The tree branch support was calculated using the ultrafast bootstrap (UFBoot) [33, 34]. Both, ModelFinder and UFBoot are utilities incorporated in IQtree.

For the morphological analysis, we built a matrix using morphological information of the identified species, considering some plesiomorphic or apomorphic characters in accordance to the proposal of Galati [35]. These were compared to our specimens (S1 Table), considering the descriptions of Mexican sand flies provided by Ibáñez-Bernal [20, 21], Ibáñez-Bernal *et al*. [18, 36, 37], and Young and Duncan [3]. We also incorporated the morphological information of two fossils species: *Pintomyia* (*Pifanomyia*) *bolontikui* [38] from the early middle Miocene (Mexican amber, Simojovel, Chiapas, Mexico) and *Micropygomyia brandaoi* [39] from the mid Miocene (Dominican amber, North Santiago, Dominican Republic), to assess the evolutionary relations among genera. The morphological information was analysed in a phylogenetic analysis using a Maximum likelihood approach in IQtree v2.2 [31]. Invariant sites were corrected with the MK+ACS model.

A total evidence phylogeny and the estimation of its divergence time were calculated using MrBayes-mpi 3.2.7–8 [40] for BioArchLinux [41]. The tree was calculated using the Fossilized Birth-Death process [42, 43], the calibration used the temporal and morphological information of the fossil species *Micropygomyia brandaoi* [39] and *Pintomyia bolontikui* [38]. The models employed for this reconstruction were obtained with Modelfinder in IQtree, limiting the search for those models exclusive to Mr. Bayes. Convergence of the Bayesian Inference (BI) analysis was verified throughout the split of frequencies of the analysis, the effective sample size (ESS), and visualization of the samples from the posterior distribution in Tracer [44].

The results of the phylogenetic analysis were summarized in a Maximum Clade Credibility (MCC) tree calculated in TreeAnnotator 2.7.0 [45] considering mean heights, and after discarding 30% of the samples. We select the MCC method, since identifies a single tree, which possesses the highest score, becoming the best tree for summarizing topological support [45]. The MCC tree was plotted against the ages and periods of the chronostratigraphic chart using the R package strap [42].

## 3. Results

### 3.1 Phlebotomine sand fly specimens analysed

We obtained specimens of phlebotomine sand fly species covering different taxa (Table 1). A total of 54 specimens, belonging to 15 species and 9 genera were analysed using PCR and DNA sequencing. We obtained a total of 99 sequences: *COI* = 32 sequences, *cytb* = 30 sequences and 18S rDNA = 37 sequences (Table 1). For the species *Br*. *mesai*, *Da*. *deleoni* and *Pa*. *shannoni*, we analysed both males and females, whereas for the remaining species, we only had female specimens available.

Using the modification of the Hoyer´s medium for the temporary mounting, we obtained favourable results for the visualization of morphological structures (S1 Fig), allowing a faster taxonomic identification for female and male phlebotomine sand flies. This method resulted to be easier, cheaper (USA $12), and faster. An advantage of the semi-permanent mounting method is that the reagents are soluble in water and, in case of need, the structures can be recovered for permanent assemblies. Since, this semi-permanent mounting use a glycerol medium, the specimens can be preserved for approximately 35 years. The use of seals in this technique is recommended and the periodic control of the state of the slide [46].

### 3.2 Morphological analysis

**Fig 2:**
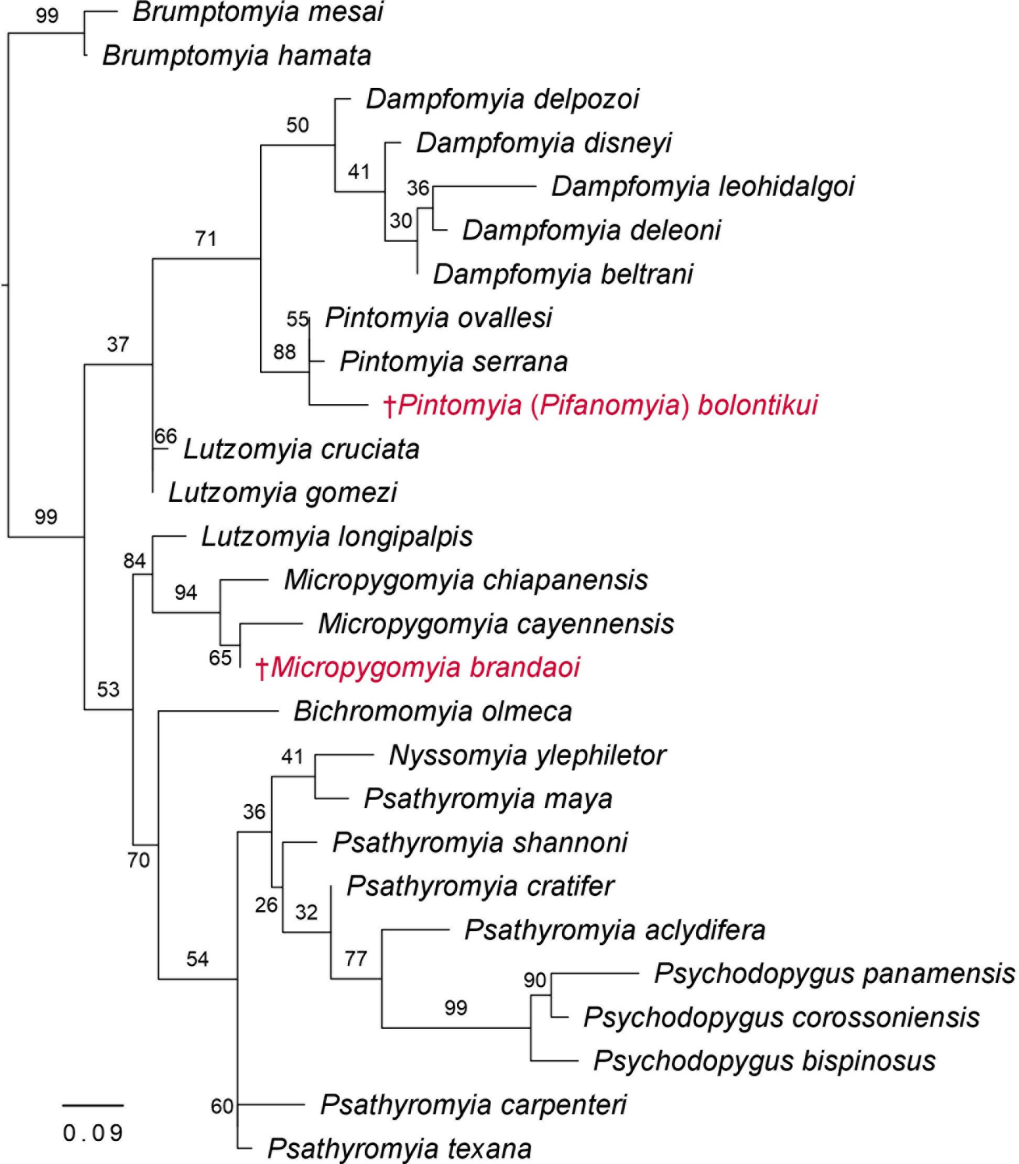
Phylogenetic relations among phlebotomine sand fly species with regard to their morphological characteristics, using Maximum Likelihood analysis. The taxa highlighted in red represent fossil species.

To create the matrix using the morphological information, we were only able to recover 48/101 characters considered by Galati [35] (S1 Table). This was due to the fact that not all taxonomic characteristics were observed when we identified the phlebotomine sand flies of this study. This occurred with the thoracic structures, since we used for molecular analysis. Besides that, in some cases the description of females or males was not available, as was the case form *Pa*. *maya* and *Da*. *disneyi*. The matrix of morphological characters included the description of 25 phlebotomine sand fly species and two fossil species (Fig 2, S1 Table).

According to the ML analysis, we observed that the morphological characteristics of the phlebotomine sand fly species grouped according to their genera and their subtribe Brumptomyiina, Lutzomyiina, Psychodopygina, Sergentomyiina, respectively, although the genera *Lutzomyia* and *Psathyromyia* are not monophyletic (Fig 2). The two fossils clustered with species of the same genera (*Pintomyia* or *Micropygomyia*, respectively) with high bootstrap support (Fig 2).

### 3.3 Sequences analysis

All the ML analyses performed for each mitochondrial gene (*COI* and *cytb*) (S2A and S2C Fig) showed bootstrap values ranging from 99 to 100% at the species level, while the gene 18S rDNA showed lower bootstrap value, except for *Br*. *mesai* (100%) and *Ps*. *panamensis* (99%) (S2B Fig). The topology was not similar for any gene, but the analysis using mitochondrial genes was better for clustering the sequences from the same species.

The *cytb* sequences showed similarities with other sequences of phlebotomine sand flies available in GenBank, ranging between 93% and 99% [10, 16, 47]. For the species *Br*. *mesai*, *Da*. *beltrani*, *Da*. *deleoni* and *Mi*. *chiapanensis*, our sequences represented the first record for the GenBank repository. The alignment contained 369 pb, a total of 228 conserved sites, 141 variable sites, and 128 parsimony-informative sites. Furthermore, 13 singletons were observed. The nucleotide diversity per site was π = 0.16521, Hd = 0.977 (21 haplotypes).

The sequences of 18S rDNA showed similarities with the following sequences available in GenBank: 99.3% with *Presattia choti* (KX356013.1); 100% with *Bichromomyia flaviscutellata* (KX356012.1); 99.28% with *Trichopygomyia trichopyga* (KX356005.1); 98.31% with *Psychodopygus panamensis* (AB288338.1); 99.52% with *Psathyromyia aragaoi* (KX356016.1); 99.28% with *Nyssomyia trapidoi* (AB288339.1); 99.76% with *Lutzomyia caballeroi* (AB638300.1); 98.55% with *Brumptomyia travassosi* (KX356006.1), and 99.8% with *Psathyromyia shannoni* (U48382.1). The sequences of 18S rDNA recorded in this study represent the first record for these species in GenBank (Table 1), except for *Pa*. *shannoni* and *Ps*. *panamensis*, which have previous records. The alignment contained 417 pb, a total of 390 conserved sites and 25 variable sites. Also, 24 parsimony-informative sites were observed.

The *COI* sequences showed a coverage between 99–100% and similarities with other *COI* sequences of phlebotomine sand flies available in GenBank ranged from 97 to 99.35% [5, 14, 15, 48, 49]. The multiple alignment contained 629 sites, and no INDEL events or stop codons were observed inside the coding region. A total of 401 conserved sites, 228 variable sites, 216 parsimony-informative sites, and 12 singletons were observed. The nucleotide diversity per site was π = 0.13363 and G + C = 0.337. With the sequences obtained, we observed 21 haplotypes, which ranged from 1 to 3 per species, with a haplotype diversity of Hd = 0.9678. The species with the higher number of haplotypes (H = 3) were *Lu*. *cruciata*, *Ny*. *ylephiletor* and *Ps*. *panamensis*.

The *COI* gene is useful to generate DNA barcodes, since it is an adequate approach for the molecular identification of several phlebotomine sand fly species and studies on genetic diversity and phylogenetic relations [5, 14, 48, 50]. Specifically, we used this approach to establish the interspecific variability distance between analysed species, which ranged from 7.9% to 19.48%. The phlebotomine sand flies *Pa*. *texana* vs *Pa*. *carpenteri* showed the lowest interspecific variability (7.9%). Contrarily, *Da*. *beltrani* vs *Pa*. *carpenteri* (19.48%); *Da*. *beltrani* vs *Ps*. *panamensis* (19.2%), and *Da*. *beltrani* vs *Pa*. *texana* (18.9%) were the species with the highest interspecific variability. The interspecific variability of the other species ranged from 12.2% to 18.42% (Table 2). We also compared the values of the intraspecific *vs* interspecific variability obtained for our sequences, and the sequences of the same species available in GenBank, to calculate the barcoding gap and to confirm the usefulness of the *COI* marker for a correct molecular identification at the species level (S3 Fig).

**Table 2:** Interspecific variability (K2P) of the *COI* sequences obtained from phlebotomine sand fly species of the states of Chiapas and Quintana Roo in Mexico.

Species	(1)	(2)	(3)	(4)	(5)	(6)	(7)	(8)	(9)	(10)	(11)	(12)
**(1) *Bi*. *olmeca***												
**(2) *Br*. *mesai***	16.8											
**(3) *Da*. *beltrani***	18.3	18.2										
**(4) *Da*. *deleoni***	13.8	15.43	12.2									
**(5) *Lu*. *cruciata***	14	15.8	17.82	16								
**(6) *Ny*. *ylephiletor***	14.3	17	18.24	15.1	16							
**(7) *Pi*. *ovallesi***	16.33	16.23	17.4	16.4	15.1	14.4						
**(8) *Pa*. *carpenteri***	15.1	15	19.48	18.42	16.23	13.8	14.8					
**(9) *Pa*. *maya***	14.8	17.55	16	15.61	14.5	14.3	18	15.54				
**(10) *Pa*. *shannoni***	16.05	17.12	18	18.31	16	14.8	17.05	14.03	16.4			
**(11) *Pa*. *texana***	15.51	13.41	18.9	18.22	15.6	15.3	15.06	7.9	14.5	15.24		
**(12) *Ps*. *panamensis***	12.7	17.03	19.2	15.2	14	14.7	18.03	12.61	16	13.5	13.07	

**Fig 3:**
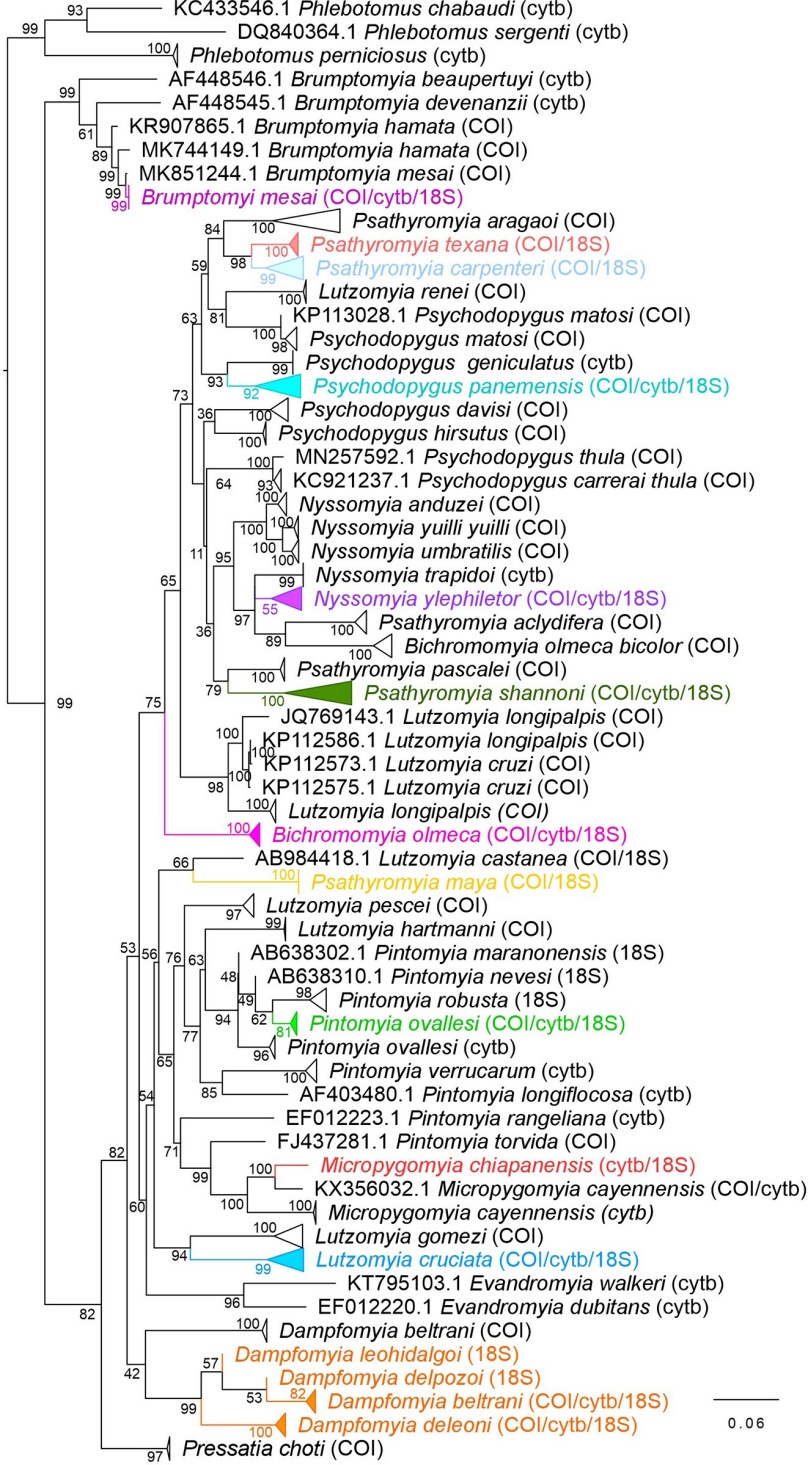
Phylogenetic relations among phlebotomine sand fly species comparing the genetic diversity of a fragment of the *COI*, *cytb* and 18S rDNA genes, using the Maximum Likelihood criterion. Coloured branches and tips correspond to sequences generated in this study. The numbers in each node indicate the bootstrap support, and the amplified genes for each species are indicated in parentheses. The triangles are collapsed branches.

The concatenated alignment had a length of 1,439 pb and a total of 214 sequences were analysed. The ML phylogenetic analysis showed that all the sequences clustered with other sequences of the same species. We were able to identify 15 species, showing high bootstrap supports (Fig 3, S4 Fig). With exception of *Da*. *beltrani* species, the sequences generated in this study (*COI*/ *cytb*/ 18S rDNA) clustered in a clade with other sequences of the same species collected in the locality of Othon P. Blanco, Quintana Roo (MK851245.1 and MK851246.1), with a bootstrap of 59%, and with other species of the genus *Dampfomyia* with a bootstrap of 98%. However, other available sequences for *Da*. *beltrani* (MK744133.1, MK744134.1 and MK744135.1) from the state of Veracruz, Mexico, clustered in a separate clade for the same species, and showing a 17.05% genetic distance from our sequences (Fig 3). A similar pattern occurred with the species *Bi*. *olmeca* and *Bi*. *olmeca bicolor*, which are considered to be a subspecies at a morphological level. However, according to our analysis, they clustered in a separated clade with a genetic distance of 14.6% (Fig 3). In *Pi*. *ovallesi*, the sequences generated in our study (*COI*/ *cytb*/ 18S rDNA) clustered with other *COI* sequences from Panama of the same species (GU001745.1, GU001746.1, MN257603.1), but not with the sequences of *cytb* gene from Colombia (AF403488.1, AF403489.1, AF403491.1) (S4 Fig). The separation between *COI* gene and *cytb* of phlebotomine sand flies of the same species was also observed in *Mi*. *cayennensis*. The other analysed species did not show this inconsistency.

According to the ML analysis of the concatenated sequences (mitochondrial and ribosomal genes), we observed that the genus *Brumptomyia* (99%), *Dampfomyia* (99%), *Evandromyia* (96%) *Micropygomyia* (100%) and *Nyssomyia* (95%) were monophyletic with a high bootstrap support. On the other hand, the genera *Lutzomyia*, *Psychodopygus*, *Pintomyia* and *Psathyromyia* were retrieved as paraphyletic. Presumably, the molecular information analysed in this work was not enough to recover the monophyly of such genera.

**Fig 4:**
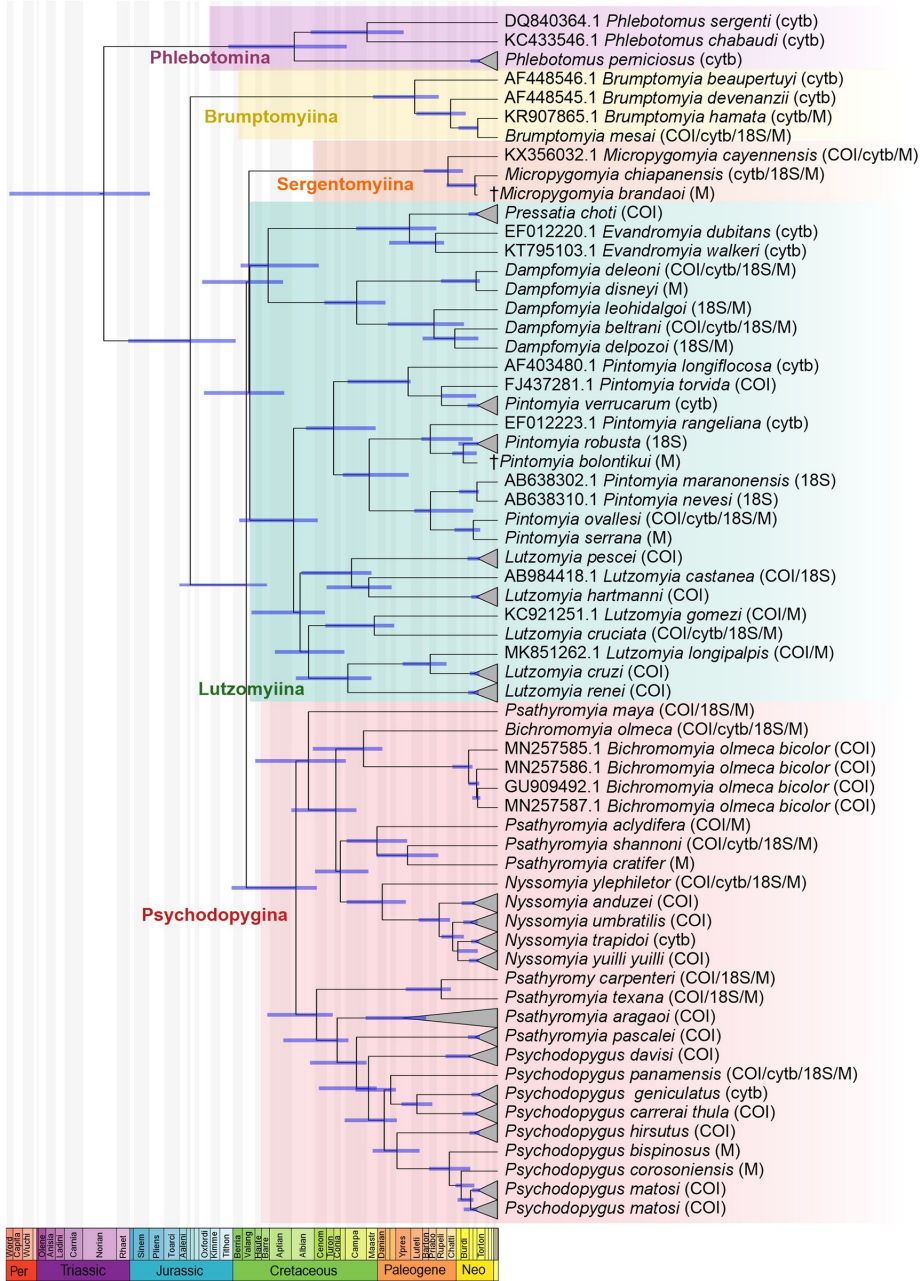
Time-calibrated phylogeny was done for phlebotomine sand flies based on the Fossilized Birth-Death process and the inclusion of divergence time estimation of each node, considering the Maximum Clade Credibility tree. In the chart, the chronostratigraphic periods are indicated. The amplified genes for each species are indicated in parentheses. The colour rectangles triangles highlight the subtribes of phlebotomine sand flies.

When we concatenated the morphological and molecular information of the phlebotomine sand fly species, we obtained similarities with previous individual analyses, which supported the taxonomic classification of some genera and species (Fig 4 and S5 Fig). These findings were consistent with the taxonomic classification proposed by Galati [11]. We also observed that several genera clustered according their Subtribes: (1) Brumptomyiina (*Brumptomyia*), (2) Sergentomyiina (*Micropygomyia*), (3) Lutzomyiina (*Lutzomyia*, *Pintomyia*, *Dampfopmyia*, *Pressatia*, *Evandromyia*) and (4) Psychodopygina (*Psathyromyia*, *Psychodopygus*, *Bichromomyia*, *Nyssomyia*). It was not possible to recover the monophyly of the genus *Psathyromyia* (Fig 4).

The inclusion of fossil evidence at morphological level allowed calibrating the estimated divergence time for the analysed phlebotomine sand flies. Several taxa have mean divergence time estimations in the Cenozoic period (between the Paleogene and Neogene periods) ranging from 10 to 63 mya. These include the phlebotomine sand flies *Br*. *mesai* (10.75 mya), *Da*. *deleoni* (11.68 mya), *Mi*. *chiapanensis* (12.31 mya), *Pi*. *ovallesi* (13.15 mya), *Da*. *beltrani* (23.24 mya), *Da*. *delpozoi* (23.24 mya), *Pa*. *carpenteri* (30.63 mya), *Pa*. *texana* (30.63 mya), *Da*. *leohidalgoi* (34.6 mya), *Pa*. *shannoni* (49.02 mya), *Ps*. *panamensis* (58.18 mya) and *Ny*. *ylephiletor* (62.79 mya) (Fig 4). On the other hand, several taxa showed an estimated older divergence time ranging from 67 to 103 mya (Cretaceous), such as *Lu*. *cruciata* (67.14 mya), *Bi*. *olmeca* (72.98 mya), and *Pa*. *maya* (103.01 mya) (Fig 4 and S6 Fig).

## 4. Discussion

An accurate taxonomic identification of phlebotomine sand flies is an important component for understanding the diversity and biology of these species, and is crucial to establish periodic entomological monitoring to provide basic information that enriches surveillance programs for vector-borne diseases [51]. Therefore, it is necessary to have appropriate and efficient tools that allow for correct taxonomic identification. In this study, we generated new molecular information on phlebotomine sand fly species distributed in endemic areas of cutaneous leishmaniasis in Mexico, validating our results with the incorporation of morphological data available for these species. We also established their phylogenetic relations and estimated their divergence time considering molecular information of other Neotropical phlebotomine sand fly species.

The phlebotomine sand fly species and the identified genera clustered according to the Subtribes Brumptomyiina, Sergentomyiina, Lutzomyiina and Psychodopygina. Although some species showed singularities, overall we found agreement between the topologies obtained by morphology (M) (Fig 2), molecular information (ML) (Fig 3), and the combined analysis (BI) (Fig 4) that included both morphological and molecular information (MCC), obtaining an integrative taxonomic classification in accordance with the molecular and morphological proposals previously described for sand fly species in other studies [8, 11].

### 4.1 Phlebotomine sand flies grouped in monophyletic genera

According to our analyses (M, ML and BI), we considered that the genus *Pintomyia* was monophyletic, which corroborates previous proposals [7, 9]. We did not find that the Series: Serrana, Ovallesi, Townsendi, and Verrucarum were monophyletic for all species. In the MCC tree (Fig 4), three main clades were observed: 1) integrated by the species *Pi*. *longiflocosa* (Townsendi), *Pi*. *torvida* (Townsendi), and *Pi*. *verrucarum* (Verrucarum); 2) included *Pi*. *rangelina* (*Pintomyia incertae sedis*), *Pi*. *robusta* (Serrana) and the fossil *Pi*. *bolontikui* (*Pintomyia incertae sedis*); and 3) composed by *Pi*. *maranonensis* (Evansi) and *Pi*. *nevesi* (Evansi), *Pi*. *ovallesi* (Evansi) and *Pi*. *serrana* (Serrana).

Similarly, the sequences of *Da*. *beltrani* clustered into two separate clades in the ML: one clade for the sequences of the state of Quintana Roo, and another for the sequences from the state of Veracruz, with a genetic distance of 17% [14]. This taxonomic separation is probably due to the fact that, although they share morphological characteristics, they probably are not of the same genus, since according to our analysis ML and BI the genus *Dampfomyia* is a monophyletic group with a 76.72 mya time of divergence (Figs 3 and 4). Hence it is suggested that the species classified as *Da*. *beltrani* distributed in Los Tuxtlas, Veracruz [14] and in Quintana Roo need a more detailed morphological review and the use of other complementary techniques, such as the geometric morphometric analysis of the wing to validate the available genetic information and discard a probable misidentification.

A similar case occurred with *Bi*. *olmeca*, which is a species morphological classified into the subspecies: *Bi*. *olmeca*, *Bi*. *olmeca bicolor* and *Bi*. *olmeca nociva* [3]. We expected that these subspecies would cluster into a monophyletic clade. Yet according to the ML analysis, the phlebotomine sand flies *Bi*. *olmeca* and *Bi*. *olmeca bicolor* clustered in separate clades with a high interspecific variability (14.6%). Considering the BI analysis, both species clustered together with a bootstrap of 65% (S5 Fig), but the estimated divergence time showed that *Bi*. *olmeca* from Mexico is an older species (72.98 mya), whereas the sequences of *Bi*. *olmeca bicolor* from Colombia (GU909492.1) and Panama (MN257587.1) appeared more recently (15.7 mya). A study based on *COI* marker showed that the *Bi*. *olmeca bicolor* species are possibly of recent evolutionary origin [52], which coincides with our results. Despite not including sequences of *Bi*. *olmeca* from Mexico, the study of Melo *et al*. [52] showed that the haplotypes of *Bi*. *olmeca* (GU001741-43) from Panama and *Bi*. *olmeca bicolor* (GU909492) from Colombia are the same species, due their low genetic variability (1%) and morphological similarities [48, 52, 53]. In our analysis (ML and BI), we now included this molecular information and confirm that in Central and South America the molecular data only correspond to the monophyletic species *Bi*. *olmeca bicolor* [48, 52–54], whereas the sequences of the *COI* and *cytb* genes from Mexico available in GenBank and the sequences generated in this study (*COI*/ *cytb*/ 18S rDNA), separate *Bi*. *olmeca* into another monophyletic group [15, 47]. Thus, we suggest that *Bi*. *olmeca* should be a valid species and not a subspecies, since genetically it is a different lineage. Complementary studies focusing on molecular and morphometric analyses are critical for the correct delimitation of species of the genus *Bichromomyia*, since species as *Bi*. *flaviscutellata*, *Bi*. *olmeca* and *Bi*. *olmeca bicolor* are of great relevance in the transmission of *Leishmania* spp. in the Americas [52].

We included DNA of male specimens of three species that coincided with sequences previously reported for the states of Quintana Roo and Veracruz [14, 15]. The inclusion of molecular information of males is important to differentiate females from closely related species, given their morphological similarity, and their potential role as a vector of *Leishmania* sp. For instance, the species of the genus *Brumptomyia*, despite of being a monophyletic genus in our analysis with a time of divergence of 45.11 mya, it included isomorphic species. Their species identification based only on morphological characteristics can be challenging, if only female specimens are included. According to our results, we found concordance between female and male sequences of *Br*. *mesai*, performing an adequate molecular and morphological identification. Complementary morphological and morphometric geometric based on wing or head, in addition to molecular information, should help to achieve correct identification of female phlebotomine sand flies. This is relevant, as this genus has been recently recorded to be infected with *Leishmania orientalis* (= *L*. *siamensis*) and *Leishmania infantum* in Ecuador and Mexico respectively, showing a potential role as a vector for transmission of these pathogens [55, 56].

### 4.2 Phlebotomine sand flies grouped in paraphyletic genera

Despite incorporating morphological and molecular information, not all the analysed species were monophyletic. We obtained similar parameters of genetic interspecific variability (7.9–19.5%) recorded for other phlebotomine sand fly species of Mexico, Colombia, Panamá, and Brazil [5, 14, 15, 48, 50, 57]. Yet, the species showing a high intraspecific variability in our study, are also considered species complexes (morphologically similar, genetically different), which could influence their taxonomic classification. We observed this in the genus *Lutzomyia*, since in the M and ML analysis the species clustered in separated clades (Figs 2 and 3). This coincides with other studies where the genus *Lutzomyia* was not considered to be monophyletic [8]. However, in the combined analysis (Fig 4), we observed that the genus *Lutzomyia* and their subgenera *Helcocyrtomyia*, *Tricholateralis* and *Lutzomyia* were monophyletic clades. The subgenus *Lu*. (*Helcocyrtomyia*) clustered the species: *Lu*. *castanea* (Serie Osornoi), *Lu*. *pescei* (Serie Peruensis) and *Lu*. *hartmanni* (Serie Sanguinaria), despite belonging to different series. The same occurred for the subgenus *Lutzomyia* (*Tricholateralis*), which grouped the species: *Lu*. *cruciata* and *Lu*. *gomezi*, and the subgenus *Lutzomyia* (*Lutzomyia*) that included the sand flies *Lu*. *longipalpis*, *Lu*. *cruzi* and *Lu*. *renei*.

This genus included anthropophilic species, such as *Lu*. *cruciata*, a species of great relevance in the transmission of *Leishmania mexicana*, and probably of *Leishmania infantum* in Mexico [56, 58]. Therefore, performing a correct classification and delimitating this genetic variation is important, given that the genetic variability could influence their capacity and competence for transmitting pathogens in some geographic areas [16]. It has been recorded that this species shows morphological changes in the shape of its head and wings, depending on its geographical distribution, which can favour the presence of genetically and biogeographically different populations [16, 59]. Previous studies have been confirming the presence of different genetic populations in *Lu*. *cruciata* in the state of Chiapas, Quintana Roo and Veracruz, Mexico [14–16]. Considering our ML analysis (S4 Fig), we detected at least three different haplotypes for *Lu*. *cruciata* using the *COI* gene (S2C Fig), and recorded the highest intraspecific variability using the *cytb* gene (2.20%) (S2A Fig). Furthermore, we observed the separation in at least two clades using the 18S rDNA gene (S2B Fig). Although, its phylogenetic classification was not clear, the analyses of M, ML and BI carried out in this study showed that *Lu*. *cruciata* and *Lu*. *gomezi* are a monophyletic clade. This is significant, since the subgenus *Lutzomyia* (*Tricholateralis*) include species of medical relevance in the transmission of *Leishmania* in other countries, such as *Lu*. *cruciata*, *Lu*. *gomezi* and *Lu*. *diabolica* [48, 58]. In Mexico, *Lu*. *cruciata* is a species recorded in 17 states, whereas the distribution of *Lu*. *gomezi* and *Lu*. *diabolica* it is more restricted [17]. However, due to the morphological similarities between the females [20], a complementary analysis of the genetic diversity and geometric morphometry of the species of this subgenus could help to delimit the species, with aid of the abundant genetic information that exists on *Lu*. *cruciata*.

We also observed that the genus *Psathyromyia* seems to be paraphyletic and that the molecular information of the analysed species did not agree with the morphological proposal. We included species of the subgenera *Forattiniella*, *Psathyromyia* and *Xiphopsathyromyia*. But in our analysis ML and BI, only the subgenus *Psathyromyia* (*Forattiniella*) is monophyletic since the species *Pa*. *texana*, *Pa*. *carpenteri*, *Pa*. *aragaoi* and *Pa*. *pascalei* clustered in similar clades (Figs 3 and 4). Unlike other species of the same subgenus, the species, *Pa*. *texana* and *Pa*. *carpenteri* showed a low interspecific variability (7.9%), suggesting that they are closely-related species with also showed similar morphology. The subgenera *Xiphopsathyromyia* was recently proposed and currently includes at least four species [18, 60], of which only *Pa*. *aclydifera* has sequences. Therefore, the taxonomic inconsistencies of this species are probably related to the lack of molecular information, and complementary information is still lacking to prove its monophyly of this subgenera. A similar scenario was observed in the phlebotomine sand fly *Pa*. *maya* described in Othon P. Blanco, Quintana Roo, where the description of the male *Pa*. *maya* remains unknown [61]. Up to now this taxon has not been well classified and is considered *Psathyromyia incertae sedis* [60]. We now extended its distribution to another locality in Quintana Roo (Noh Bec) and generated novel molecular information. However, it is necessary to incorporate additional information to delimit its taxonomic classification, since according to our analyses, it clustered with different species (Figs 3 and 4), showing a divergence time of 103.01 mya.

The phylogenetic classification of *Pa*. *shannoni* with regard to other species of the genus *Psathyromyia*, showed inconsistent data in the different analyses: in the ML analysis it clustered with *Pa*. *pascalei* (Fig 3), and in the BI analysis it clustered with *Pa*. *cratifer* and *Pa*. *aclydifera* (Fig 4). The phlebotomine sand fly *Pa*. *shannoni* is included in the Shannoni complex, which groups at least six species. It has recently been proposed that due its high intraspecific variability, this species could be separated into different genetic linages according to their geographical distribution [62]. Although in this study we did not include other species of the Shannoni complex, the sequences generated in our study were similar to other sequences recorded in Othon P. Blanco, Quintana Roo [15, 49]. The addition of a new haplotype from the locality San Antonio Buenavista, Chiapas, now shows an intraspecific variability of 1.26%, as compared to the sequences of Quintana Roo. Yet, since the intraspecific variability observed in both haplotypes was within the accepted limits for species delimitation for the Shannoni complex (0.3–4%), the specimens of *Pa*. *shannoni* collected in this study belong to the Mexican lineage [62].

The use of the 18S rDNA gene was not helpful in resolving phylogenetic relations at the genus and subgenus level as we expected. However, the incorporation of other nuclear genes, such as 28S rDNA, could provide complementary information at the subgenera level to resolve these inconsistences [7]. Clearly, a more exhaustive taxonomic revision is necessary for the species included in the genus *Lutzomyia* and *Psathyromyia* and their subgenera *sensu* Galati (2019) [11].

### 4.3 Estimated divergence time for sand fly species

Our analysis showed that some phlebotomine sand flies likely diverged during the Carboniferous period. The divergence of the subfamily Phlebotominae could have occurred between the Jurassic and the Cretaceous periods, supporting a hypothetical phlebotomine-like ancestor proposed to have emerged at similar time [1]. Despite that several fossils have been describe in the New World, all are specimens from Dominican and Mexican amber of the genera *Pintomyia* (13), *Micropygomyia* (3), and *Psathyromyia* (1) [60]. For that reason, we only included the morphological characteristic of one specimen of the genus *Pintomyia* and another for *Micropygomyia*. We exclude the *Psathyromyia* fossil since, according to our results, it is not a monophyletic genus.

Additional fossil evidences showed that the phlebotomine sand flies of the Old World (Lebanon) dated approximately 120 mya. Therefore the evolution of the subfamily Phlebotominae could have been influenced by the separation of Pangaea, dividing phlebotomine sand fly species in the Old World and New World [1].

Two main hypotheses have been proposed for the possible origin of phlebotomine sand flies. One hypothesis assumes that phlebotomine sand flies evolved in the Palaearctic ecozone during the Cretaceous period, and the species were isolated due the split of Pangaea causing an independent evolution, which resulted in the origin of the genera of the Old World (during the Eocene), and the genera of the New World (during the Oligocene). A second hypothesis assumes that the phlebotomine sand flies existed in Gondwana before the continental separation, due to their morphological similarities between current and fossils phlebotomine sand fly taxa [1].

In the time-calibrated phylogeny, we can highlight that the analysed phlebotomine sand fly species were divided into species with older and relatively recent divergence times. Some sand fly species with the oldest estimated divergence times showed higher genetic variability (mentioned above), hindering that their phylogenetic relationships be resolved. For instance, the phlebotomine sand fly species: *Bi*. *olmeca* (72.98 mya), and *Lu*. *cruciata* (67.98 mya) showed an estimated divergence time from the Cretaceous periods. Conversely, some species with a less divergence time have a more restricted distribution and their genetic variability is more conserved, supporting the hypothesis of radiation of phlebotomine sand fly species throughout the Neotropics [1].

For example, the species of the genus *Pintomyia* is a monophyletic genus that includes species with the lowest divergence time, which ranging from 10 mya to 48 mya (Fig 4 and S6 Fig). Despite that we included a fossil sand fly (*Pi*. *bolontikui*) of this genus; this species was not the oldest species (18.62 mya), as according to the BI analysis other ancestors probably date to the Cretaceous (89.31 mya) (Fig 4 and S6 Fig). The same was observed in the genus *Micropygomyia* with a divergence time of 26.97 mya, while the fossil species (*Mi*. *brandaoi*) showed a divergence of 12.31 mya. In the case of sand fly species such as *Pa*. *texana* and *Pa*. *carpenteri* the divergence time is older with 30.63 mya. However, *Pa*. *texana* has a limited distribution in Mexico and USA, whereas *Pa*. *carpenteri* has a wide distribution from Mexico to Colombia [60]. According to the ML analysis (Fig 3 and S4 Fig) the sequences from the Mexican southeast and Colombia clustered in a clade with a bootstrap support of 98%, confirming that they are the same species. If we consider that the Isthmus of Panama formation dates from 23 to 3.5 mya [63], the divergences time obtained in the BI analysis with our sequences for *Pa*. *carpenteri* was 30.63 mya (Fig 4 and S6 Fig) suggests that this species probably diverged in Mexico and then it spread to Colombia. It is important to highlight that this is only a hypothesis that tries to make an approximation of the divergence times for some Mexican sand fly species. However, it is necessary to include more morphological and molecular information as well as more fossil evidence from other taxa, in order to have more precise calibration points to strengthen and test the divergence time estimation. However, our results support the hypothesis that evolution of some phlebotomine sand fly species distributed in Mexico could have occurred during the Cretaceous period.

Our study provides novel molecular information for 15 phlebotomine sand fly species from different areas of Mexico, contributing to the genetic inventory and phylogenetic relationships of species of the subfamily Phlebotominae from the Neotropic areas. According to our results, the mitochondrial genes are appropriate markers for the molecular identification of phlebotomine sand fly species. However, it is necessary to conduct adequate morphological identifications and include complementary characters and additional molecular information, since the boundaries of intraspecific variability for each phlebotomine sand fly species remains unknown. Thus, it is necessary to increase the geographic sampling to include information from closely related species and analyse their genetic variability (intra- and interspecific). The use of mitochondrial markers such as *COI* and *cytb*, has proven useful for the molecular identification of phlebotomine sand flies, allowing to discriminate between species, even if they are cryptic or closely related species. The incorporation of nuclear genes in future molecular studies will increase the significance of phylogenetic inferences at subgenera level.

## Supporting information

S1 Table List of morphological characteristics for 27 phlebotomine sand fly species. (XLSX)

S1 Fig Images of different structures of taxonomic relevance obtained using temporary mounting. (a) wing of *Psathyromyia texana*; (b) flagellomere and ascoids of *Dampfomyia deleoni*; cibarium: (c) *Psathyromyia shannoni*, (d) *Psathyromyia texana*, (e) *Dampfomyia deleoni*, (f) spermathecae of *Psathyromyia shannoni*, and (g) male genitalia of *Brumptomyia mesai*.
(TIF)

S2 Fig **A.** Phylogenetic relations among sand fly species from Mexico to compare the genetic diversity of a partial fragment of the *cytb* gene, using the Maximum Likelihood analysis. **B.** Phylogenetic relations among sand fly species from Mexico to compare the genetic diversity of a partial fragment of the 18S rDNA gene, using the Maximum Likelihood analysis. **C.** Phylogenetic relations among sand fly species from Mexico to compare the genetic diversity of a partial fragment of the *COI* gene, using the Maximum Likelihood analysis. The numbers in each node indicate the bootstrap support.
(ZIP)

S3 Fig Barcoding Gap of sand fly species calculated using Kimura 2-parameters substitution model. The frequency (A) and density (B) of calculated intra- and interspecific genetic distances are depicted. Dashed red lines show mean values. Our sequences and some GenBank sequences of the same species are included.
(TIF)

S4 Fig Complete phylogenetic relations among different sand fly species to compare the genetic diversity of a partial fragment of the *COI*, *cytb* and 18S rDNA gene, using the Maximum Likelihood analysis. The colours highlight the sequences generated in this study; the numbers in each node indicate the bootstrap support. The black triangles are collapsed branches.
(TIF)

S5 Fig Phylogenetic relations between twelve genera of sand fly species according to their molecular (*COI*/ *cytb*/ 18S rDNA) and morphologic (M) information, using Maximum Likelihood. The colours represent the genera analysed and their species, the numbers in each node indicate the bootstrap support, and the symbol + highlight the species fossils.
(TIF)

S6 Fig Time-calibrated phylogeny for phlebotomine sand flies based on the Fossilized Birth-Death process and the inclusion of divergence time estimation of each node, considering the Maximum Clade Credibility (MCC) tree. (TIF)
