# Supplementary figures and images for: Phylogenetic relations among Mexican phlebotomine sand flies (Diptera: Psychodidae) and their divergence time estimation
Source: PLoS One. 2023 Jun 29;18(6):e0287853. doi: 10.1371/journal.pone.0287853 (PMC10309607; doi:10.1371/journal.pone.0287853)

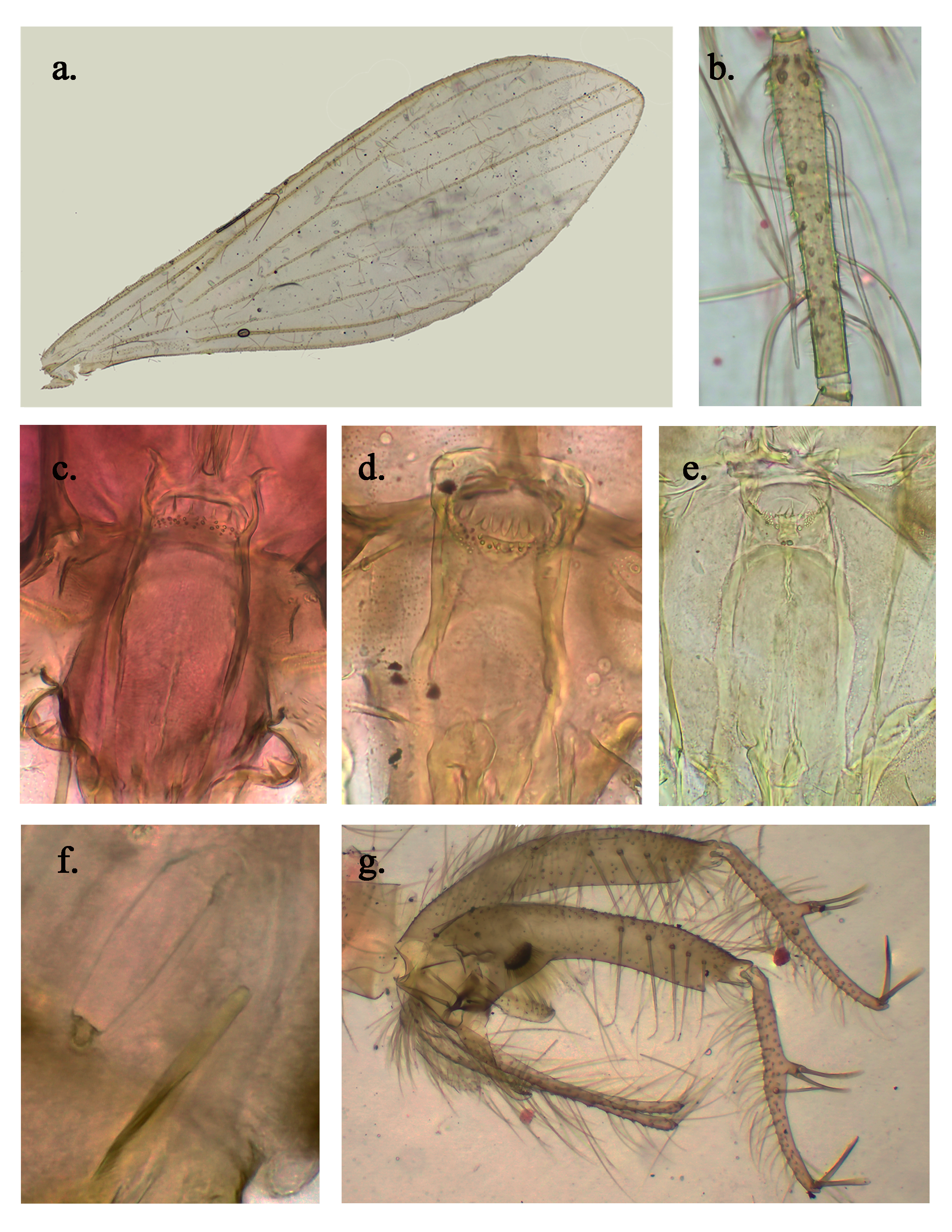

Supplement: S1 Fig — (a) wing of Psathyromyia texana; (b) flagellomere and ascoids of Dampfomyia deleoni; cibarium: (c) Psathyromyia shannoni, (d) Psathyromyia texana, (e) Dampfomyia deleoni, (f) spermathecae of Psathyromyia shannoni, and (g) male genitalia of Brumptomyia mesai. (TIF) [file pone.0287853.s002.tif]

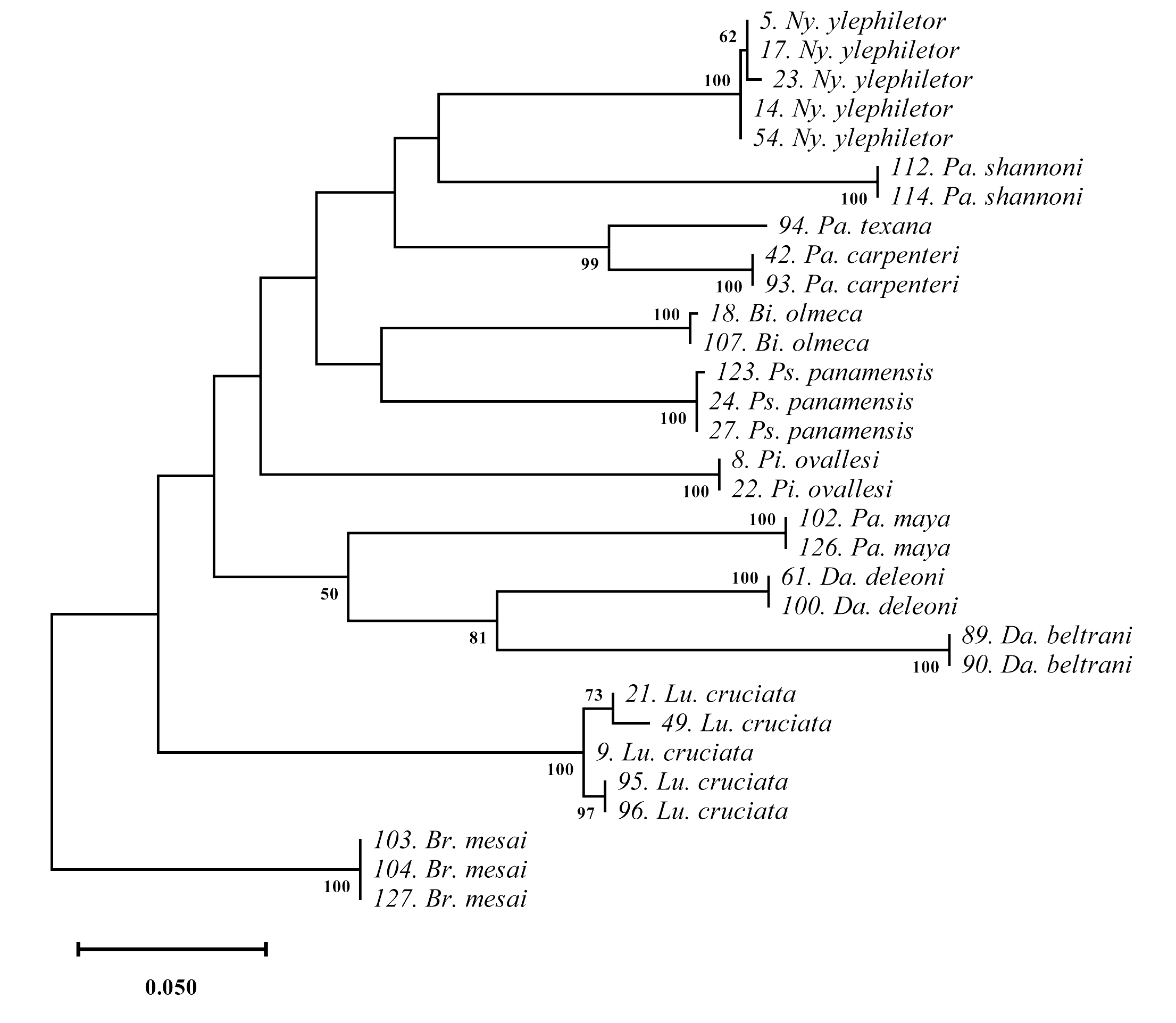

Supplement: S2 Fig — A. Phylogenetic relations among sand fly species from Mexico to compare the genetic diversity of a partial fragment of the cytb gene, using the Maximum Likelihood analysis. B. Phylogenetic relations among sand fly species from Mexico to compare the genetic diversity of a partial fragment of the 18S rDNA gene, using the Maximum Likelihood analysis. C. Phylogenetic relations among sand fly species from Mexico to compare the genetic diversity of a partial fragment of the COI gene, using the Maximum Likelihood analysis. The numbers in each node indicate the bootstrap support. (ZIP) [file pone.0287853.s003.zip › S2C_Fig.tif]

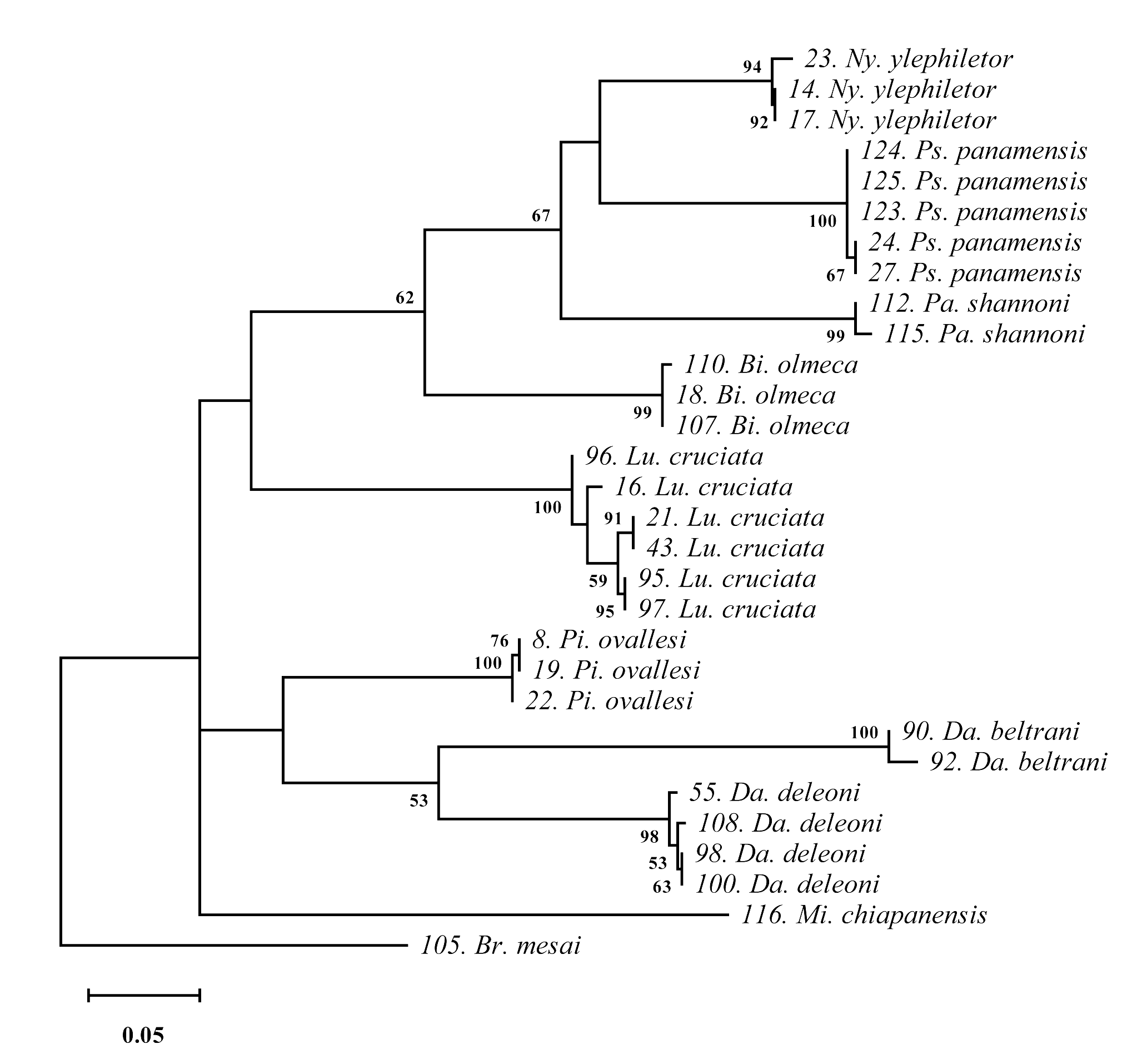

Supplement: S2 Fig — A. Phylogenetic relations among sand fly species from Mexico to compare the genetic diversity of a partial fragment of the cytb gene, using the Maximum Likelihood analysis. B. Phylogenetic relations among sand fly species from Mexico to compare the genetic diversity of a partial fragment of the 18S rDNA gene, using the Maximum Likelihood analysis. C. Phylogenetic relations among sand fly species from Mexico to compare the genetic diversity of a partial fragment of the COI gene, using the Maximum Likelihood analysis. The numbers in each node indicate the bootstrap support. (ZIP) [file pone.0287853.s003.zip › S2A_Fig.tif]

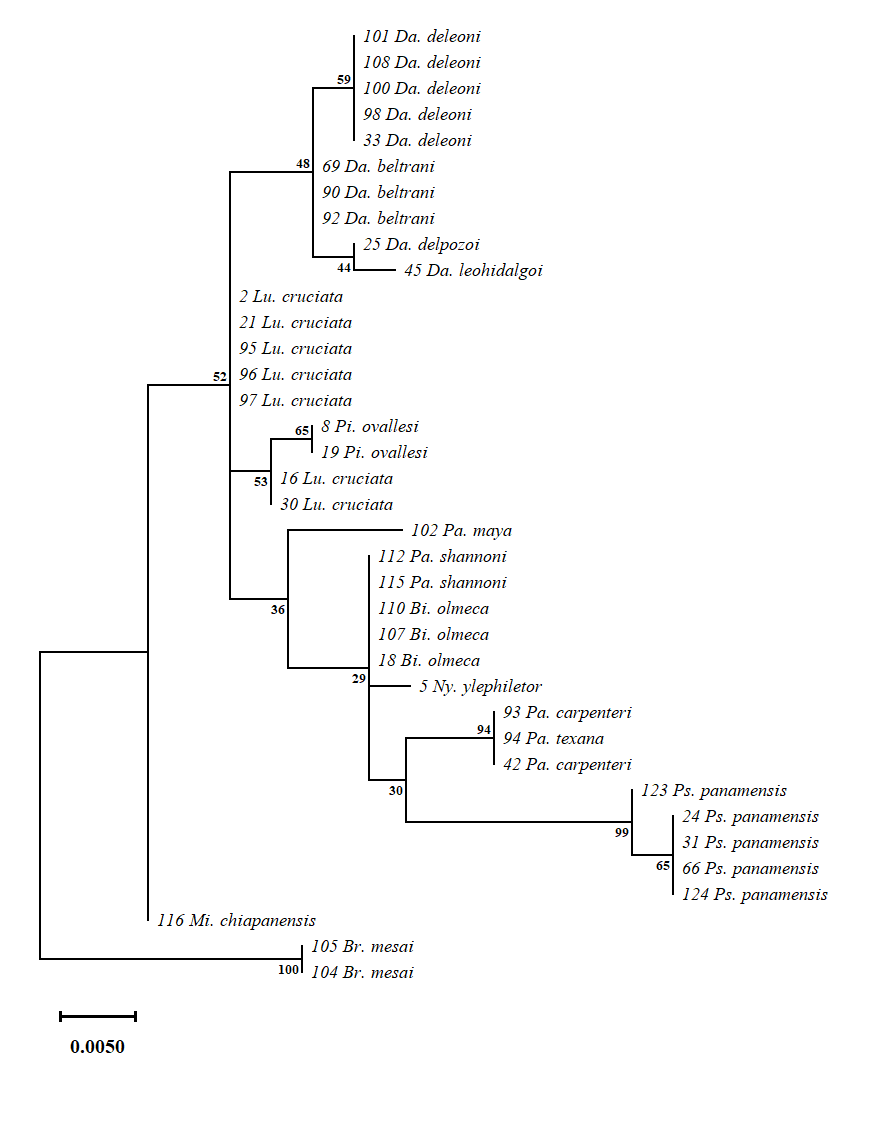

Supplement: S2 Fig — A. Phylogenetic relations among sand fly species from Mexico to compare the genetic diversity of a partial fragment of the cytb gene, using the Maximum Likelihood analysis. B. Phylogenetic relations among sand fly species from Mexico to compare the genetic diversity of a partial fragment of the 18S rDNA gene, using the Maximum Likelihood analysis. C. Phylogenetic relations among sand fly species from Mexico to compare the genetic diversity of a partial fragment of the COI gene, using the Maximum Likelihood analysis. The numbers in each node indicate the bootstrap support. (ZIP) [file pone.0287853.s003.zip › S2B_Fig.tif]

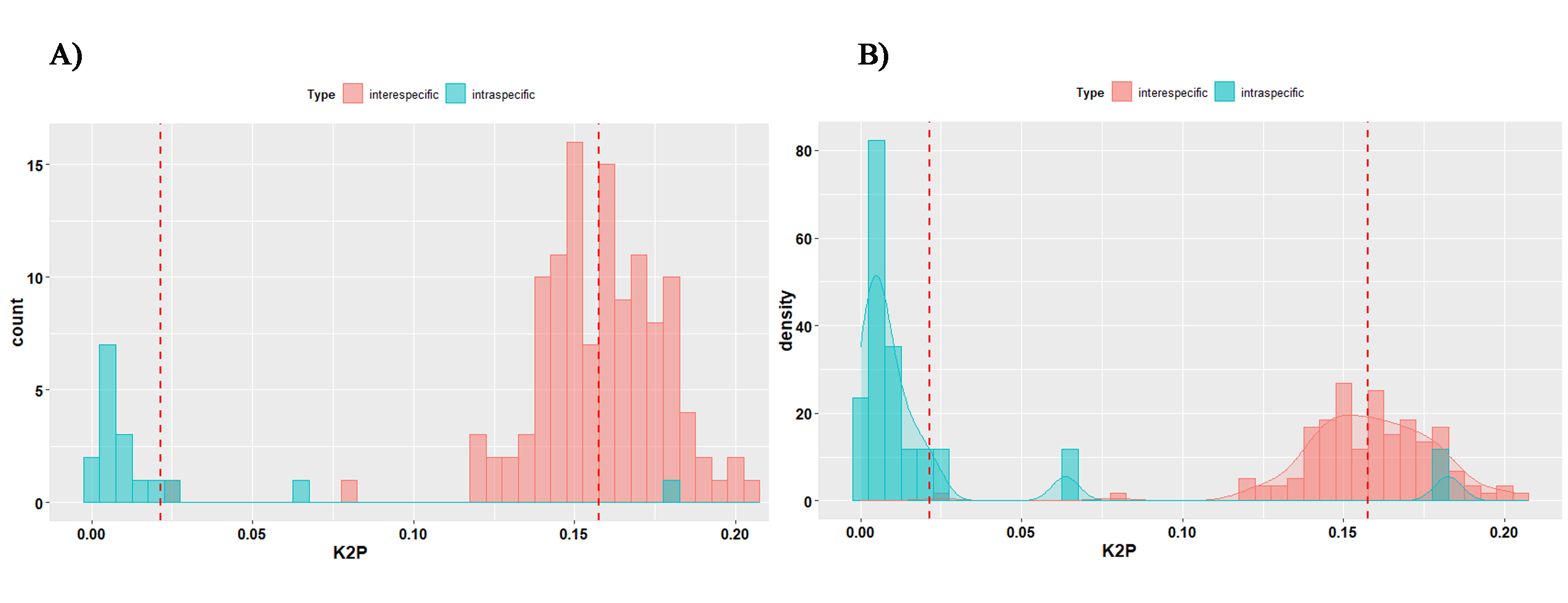

Supplement: S3 Fig — The frequency (A) and density (B) of calculated intra- and interspecific genetic distances are depicted. Dashed red lines show mean values. Our sequences and some GenBank sequences of the same species are included. (TIF) [file pone.0287853.s004.tif]

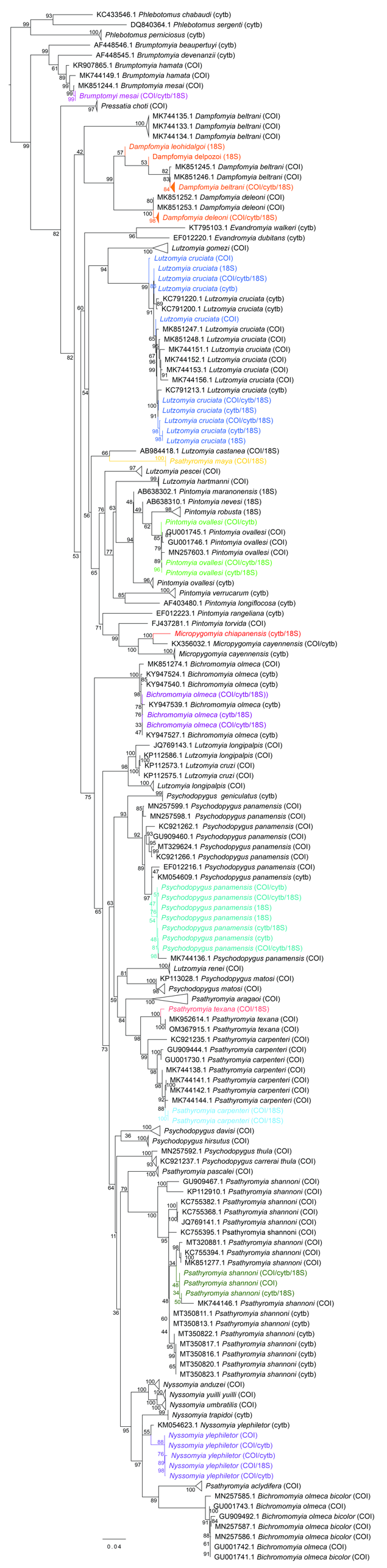

Supplement: S4 Fig — The colours highlight the sequences generated in this study; the numbers in each node indicate the bootstrap support. The black triangles are collapsed branches. (TIF) [file pone.0287853.s005.tif]

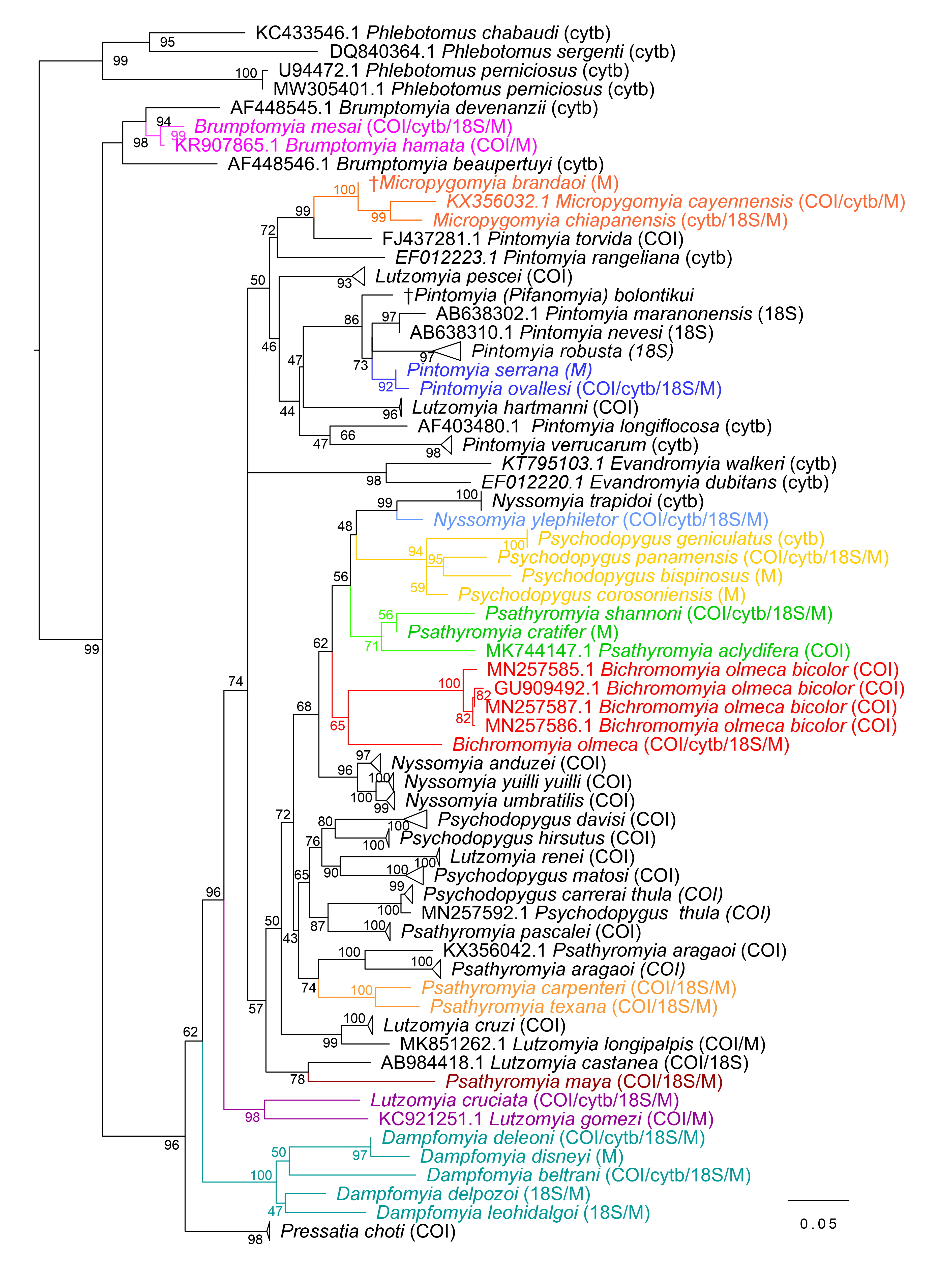

Supplement: S5 Fig — The colours represent the genera analysed and their species, the numbers in each node indicate the bootstrap support, and the symbol + highlight the species fossils. (TIF) [file pone.0287853.s006.tif]
